# miR-2053 inhibits the growth of ovarian cancer cells by downregulating SOX4

**DOI:** 10.1515/med-2023-0667

**Published:** 2023-05-23

**Authors:** Xin Huang, Wen Zhang, Xiumin Shen, Sai Ma, Lili Liu

**Affiliations:** Department of Obstetrics, The First Affiliated Hospital of Jinzhou Medical University, Jinzhou, Liaoning 121000, P.R. China; Departments of Haematology, The First Affiliated Hospital of Jinzhou Medical University, Jinzhou, Liaoning 121000, P.R. China; Department of Obstetrics, The First Affiliated Hospital of Jinzhou Medical University, 2 Renmin Street, Guta, Jinzhou, Liaoning 121000, P.R. China

**Keywords:** miR-2053, SOX4, ovarian cancer

## Abstract

Ovarian cancer is one of the major gynaecological malignancies and a leading cause of cancer-related deaths worldwide. Dysregulation of miR-2053 has been reported in numerous types of cancer; however, its function in ovarian cancer remains largely unknown. In our study, the roles of miR-2053 during the development of ovarian cancer were investigated. miR-2053 expression was examined in ovarian cancer specimens and cells. Furthermore, the detailed functions and downstream targets of miR-2053 were identified. Briefly, the levels of miR-2053 were assessed in ovarian cancer tissues and paired non-cancerous samples, as well as in ovarian cancer cells using reverse transcription-quantitative polymerase chain reaction. The proliferation of cells was determined by cell counting kit-8 kit, and the levels of PCNA were also examined using immunostaining. Cell migration and invasion were evaluated using Transwell assay, and E-cad expression was assessed by immunostaining. In addition, cell apoptosis was determined by flow cytometry, and the expression of cleaved caspase-3 was examined using western blotting. The results revealed the downregulation of miR-2053 in ovarian cancer tissues and cells. Moreover, miR-2053 mimics suppressed the proliferation, migration, and invasion of ovarian cancer cells, while cell apoptosis was promoted. In addition, SOX4 was a putative downstream molecule of miR-2053 in ovarian cancer. Furthermore, SOX4 is involved in miR-2053-regulated growth and metastasis of ovarian cancer cells. In summary, miR-2053 and its novel target SOX4 could serve essential roles during tumour development of ovarian cancer, more importantly, miR-2053/SOX4 axis may be novel candidate for targeted therapy for patients with ovarian cancer.

## 1 Introduction

Ovarian cancer is one of the major gynaecological malignancies and a leading cause of cancer-related deaths in females globally [1,2]. The mortality rate of ovarian cancer is high due to the lack of efficient diagnostic methods at early tumour stages; therefore, the prognosis remains poor [1,2,3]. Chemotherapy is broadly used for patients with ovarian cancer, but drug resistance is very common [4,5]. Furthermore, the recurrence rates of ovarian cancer are extremely high, which also contributes to the high mortality rate of this disease [2,3,4,5]. Recently, the underlying molecular mechanisms in the pathogenesis of ovarian cancer have been widely investigated, and novel candidates have been reported for the diagnosis and targeted treatment of this disease [3,4,5]. Among these, microRNAs (miRNAs) have been implicated in the progression of ovarian cancer [6,7,8].

miRNAs are a group of endogenous non-coding RNAs and are ∼22 nt in length. miRNAs are novel targets of other types of non-coding RNAs such as lncRNAs and circRNAs [9,10]. Previous studies have revealed that miRNAs could serve essential roles through binding to 3′-UTR of corresponding target mRNAs [11,12,13,14]. Aberrant levels of miRNAs have been detected in cancer patients, which is associated with tumourigenesis [9,13,14,15], and miRNAs were considered promising biomarkers for tumour development [16]. For instance, miR-93 could promote the growth of glioma by targeting PI3K/Akt pathway [13]. Moreover, miR-15 and miR-16 were able to enhance cell apoptosis via BCL2 signalling [14]. At present, the detailed roles and molecular mechanisms of miRNAs in tumour development in ovarian cancer remain largely unknown and require further study. Recently, dysregulation of miR-2053 has been detected in hepatocellular carcinoma, and overexpressed miR-2053 was able to suppress the progression of cancer cells by targeting PI3K/Wnt/b-catenin signalling [17]. However, its expression profile and detailed function in ovarian cancer are still not clear.

Dysregulation of miR-2053 has been reported in numerous types of cancer, and it is involved in the progression of tumour. The aim of this study was to explore the regulatory functions of miR-2053 using gain- and loss-of-function experiments. In the present study, downregulation of miR-2053 was observed in ovarian tissues and cells. Furthermore, cell proliferation, migration, and invasion were inhibited following the transfection with miR-2053 mimics, whereas cell apoptosis was promoted. In addition, SOX4 could be a promising target of miR-2053 in ovarian cancer. Moreover, SOX4 was involved in miR-2053-modulated ovarian cancer growth. Taken together, miR-2053/SOX4 signalling could regulate the proliferation, apoptosis, migration, and invasion of ovarian cancer cells, and this novel pathway may be a candidate target for the optimized treatment of this disease.

## 2 Materials and methods

### 2.1 Clinical specimens

Fifty ovarian cancer tissues and matched adjacent normal specimens were obtained from our hospital from March 2015 to June 2016. The patients underwent surgery at the Department of Obstetrics and Gynecology, and none of the participants received radio- or chemo-therapy and surgical or biological treatment prior to the operation. The mean age was 55.8 ± 10.2 years; 24 cases were diagnosed with stage I–II ovarian cancer, while the rest were diagnosed with stage III–IV; lymph node metastasis was found in 29 participants. The follow-up of patients was carried out every 3 months for 3 years post-surgery. Then, patient biopsies were sectioned and stored at −80°C until further use. The biopsies were examined and diagnosed by three independent pathologists. Overall survival of the patients was interpreted by Kaplan–Meier test. The protocol was reviewed and approved by the Ethics Committee of the First Affiliated Hospital of Jinzhou Medical University. Written informed consents were obtained from all patients, and information of clinical samples was anonymized.

### 2.2 Cell culture

Human ovarian cancer cells (OVCAR-3, A2780, COC1, and SKOV3) together with IOSE 80 cells were purchased from American Type Culture Collection (Manassas, VA, USA) in June 2018. Cells were cultured using RPMI-1640 medium (GE Healthcare Life Science) supplemented with 10% foetal bovine serum (FBS) (GE Healthcare Life Science), 1× non-essential amino acids (Sigma Aldrich, Irvine, UK), 100 µL/mL (v/v) penicillin–streptomycin (Sigma Aldrich), 1 mM sodium pyruvate (Sigma Aldrich), and 1 µL/mL bovine insulin (Sigma Aldrich). All the cells were kept in a humid incubator supplied with 5% CO_2_ at 37°C. All experiments were performed with mycoplasma-free cells.

### 2.3 Transfection

miR-2053 mimics and inhibitors, as well as negative control (miR-NC) containing non-targeting sequence, were purchased from Genepharm Co. Ltd. (Shanghai, China). To produce cells overexpressing SOX4, wildtype (LV-SOX4) and mutant (LV-NC) sequences were amplified using PCR. Then, the fragments were inserted into pCMV vector provided by the Chinese Academy of Sciences (Changchun, China). 0.3 mg/mL of plasmids were used, and all transfections were performed using Lipofectamine^®^2000 (Invitrogen; Thermo Fisher Scientific, Inc.). Up- or down-regulation of genes was confirmed using reverse transcription-quantitative polymerase chain reaction (RT-qPCR). Following 12 h, culture supernatant was replaced with fresh culture medium with 10% FBS.

### 2.4 RNA extraction and RT-qPCR

Total RNA was isolated from clinical samples and cell lines by TRIzol^®^ reagent (Invitrogen; Thermo Fisher Scientific, Inc.). Extracted RNA was reverse transcribed using PrimeScript™ RT kit (Takara Biotechnology Co., Ltd., Dalian, China). Subsequently, quantitative PCR was carried out using SYBR Green PCR Master Mix (TaKaRa Biotechnology Co., Ltd.), and all the amplification was performed using ABI 7500 system (Thermo Fisher Scientific, Inc.). TaqMan MicroRNA Assay (Applied Biosystems; Thermo Fisher Scientific, Inc.) was used to examine the levels of miR-2053, followed by qPCR using the Applied Biosystem 7500. U6 and GAPDH were used as control genes to normalize the expression. Forward and reverse primers used for qPCR were as follows: miR-2053, 5′-GUGUUAAUUAAACCUCUAUUUAC-3′ and 5′-GUAAAUAGAGGUUUAAUUAACAC-3′; PCNA, 5′-CATGGTGAAACCCCGTCTCTACTA-3′ and 5′-GAGCACTTAGGCAATTTTGGTGAT-3′; E-cad, 5′-GGTTATTCCTCCCATCAGCT-3′ and reverse, 5′-CTTGGCTGAGGATGGTGTA-3′; SOX4, 5′-CGCGGAATTTGGGCGCCCGCCGAGCCGAG-3′ and reverse, 5′-CGCGGGATCCCCTTCAGTAGGTGAAAACCAG-3′; GAPDH, 5′-GCAAGAGCACAAGAGGAAGA-3′ and reverse, 5′-ACTGTGAGGAGGGGAGATTC-3′. The PCR program was as follows: denaturation at 95°C for 5 min, 45 cycles of 95°C for 15 s, 60°C for 20 s, and 72°C for 10 s, followed by the final extension at 72°C for 30 s.

### 2.5 Immunofluorescence staining

Transfected cells were fixed in ice-cold acetone (Sigma­Aldrich, Poole, UK) for 10 min. Subsequently, cells were washed three times using phosphate-buffered solution (PBS) and incubated in blocking reagent for 1 h, followed by incubation in primary antibodies against PCNA (1:100; cat. no. 13-3900; Invitrogen) or E-cad (1:100; cat. no. ab40772; Abcam) at 4°C overnight. In the following day, cells were then washed with PBS and incubated with Alexa-Fluor 568-conjugated secondary antibody (1:2,000, Molecular Probes, Eugene) in the dark for 1 h. The secondary antibody-only group was used as a negative control. Cell nuclei were counter-stained with DAPI (Vector Laboratories, Peterborough, UK). Then, cells were washed with PBS and mounted using the Mowiol reagent containing 10% Mowiol D488 (Calbiochem, Nottingham, UK). Stained cells were checked under the Leica DMLB Microscope. Images were captured by a CCD camera (Cool-SNAP-Pro; Media Cybernetics, USA) and analysed using Image-Pro Plus (version 6.0; Media Cybernetics, USA).

### 2.6 Western blot analysis

Protein samples were isolated using RIPA buffer (Beyotime Institute of Biotechnology, Shanghai, China). The concentration of extracted samples was evaluated by a BCA kit (Beyotime Institute of Biotechnology). An equal amount (∼40 μg) of samples was separated using 10% sodium dodecyl sulfate-polyacrylamide gel electrophoresis gels, and protein was then transferred to a nitrocellulose membrane (EMD Millipore, Billerica, MA, USA). Then, membranes were blocked with PBS containing 5% non-fat milk for 1 h, followed by incubation in primary antibodies against cleaved caspase-3 (1:1,000; cat. no. ab32351; Abcam), SOX4 (1:1,000; cat. no. ab243041; Abcam), or GAPDH (1:1,000; cat. no. ab9485; Abcam) in a cold room overnight. In the next day, membranes were then incubated with horse radish peroxidase-conjugated anti-mouse (1:2,000; cat. no. 7076; Cell Signalling) or -rabbit IgG (1:2,000; cat. no. 7074; Cell Signalling) at room temperature for 1 h. Blots were detected using an ECL kit (Pierce Biotechnology; Thermo Fisher Scientific, Inc). The density of bands was quantified using ImageJ (NIH, Bethesda, MD, USA).

### 2.7 Cell counting kit-8 (CCK-8) assay

Transfected cells were seeded on a 96-well plate at a density of 6 × 10^4^ cells per well. In brief, the proliferation of cells was determined on Days 1–4. Subsequently, 10 μL of CCK-8 solution (Dojindo Molecular Technologies, Inc., Kumamoto, Japan) was added to each well. Furthermore, the cells were incubated for an additional 2 h, and then, the absorbance at 450 nm was read on a microplate reader (Bio-Rad Laboratories, Inc., Hercules, CA, USA).

### 2.8 Transwell migration and invasion assay

The migratory and invasive abilities of the cells were evaluated by Transwell assay. Briefly, the suspension of 4 × 10^5^ cells containing no FBS was seeded into the upper chamber with a pore size of 8 µm (BD Biosciences, Franklin Lakes, NJ, USA). In addition, for cell invasion, cells were seeded onto the upper chamber, which is pre-coated with Matrigel^®^ (Sigma-Aldrich, St. Louis, MO, USA). Subsequently, 500 µL culture media containing 10% FBS was added into the lower chamber. After the incubation for 2 days, non-migratory or -invasive cells were removed using a cotton bud. The cells within the lower chamber were fixed using ice-cold methanol for 15 min and then stained with crystal violet (0.5%; Sigma Aldrich). Stained cells were counted in 10 randomly selected fields under an inverted light microscope (magnification ×100, Olympus Corporation, Tokyo, Japan).

### 2.9 Assessment of cell apoptotic rate

Transfected cells with a density at 4 × 10^5^ cells/well were inoculated onto a six-well plate. Briefly, the cell suspension was centrifugated by low-speed centrifugation (1,000 rpm) at 4°C for 10 min. Then, cell pellets were washed with PBS and well suspended, then fixed in ice-cold ethanol (70%, Sigma Aldrich), and then stored in the cold room for 2 days. Prior to flow cytometry, cells were lysed, centrifugated, and re-suspended. The Annexin V-fluorescein isothiocyanate (FITC)/propidium iodide (PI) apoptosis kit (BD Biosciences, Franklin Lakes, NJ, USA) was used to determine the apoptotic rate of ovarian cancer cells. In PI (Sigma-Aldrich, USA) staining buffer containing PI (50 µL/mL) and RNase A (250 µL/mL). Briefly, transfected cells were well suspended and subsequently stained with 10 μL Annexin V-FITC and 5 μL PI in the dark at 4°C for 30 min. Cell apoptotic rate was determined using a flow cytometer (BD Biosciences, USA), and results were interpreted by Flowjo (version 10.6, Flowjo LLC, USA).

### 2.10 Bioinformatics analysis and luciferase assay

TargetScan (www.targetscan.org/) was used to predict the novel targets of miR-2053. For the luciferase reporter assay, wild-type (SOX4-WT) sequence of the 3′-UTR on SOX4 carrying putative binding sites of miR-2053 was obtained from Shanghai GenePharma Co., Ltd. and was then integrated into the pmirGLO Dual-Luciferase miRNA Target Expression Vector (Promega Corporation). QuikChange Multi Site-Directed Mutagenesis Kit (Stratagene; Agilent) was used to generate SOX4-MUT reporter carrying mutant miR-2053-binding sequence. The plasmids were co-transfected with miR-NC or miR-2053 mimics into DH5α competent cells. Luciferase activity was examined 48 h after transfection by a Dual-Luciferase Reporter Assay kit (Promega Corporation). The activity of firefly luciferase was normalized using *Renilla* luciferase.

### 2.11 Statistics analysis

All the data were presented as mean ± standard error. Data analyses were carried out using SPSS (version 24.0; Chicago, USA). Statistical significance was examined using Student’s *t*-test or one-way analysis of variance (ANOVA). Tukey test was conducted as a post hoc test following ANOVA. The relationship between RNA expression was evaluated using Pearson’s correlation test. Receiver operating characteristic (ROC) curve analyses were performed to investigate the power of miR-2053 levels on differentiating ovarian cancer tissues and adjacent normal specimens, cases with and without metastasis, and patients with low grade (I–II) and high grade (III–IV).

## 3 Results

### 3.1 The levels of miR-2053 were decreased in GC tissues

**Figure 1:**
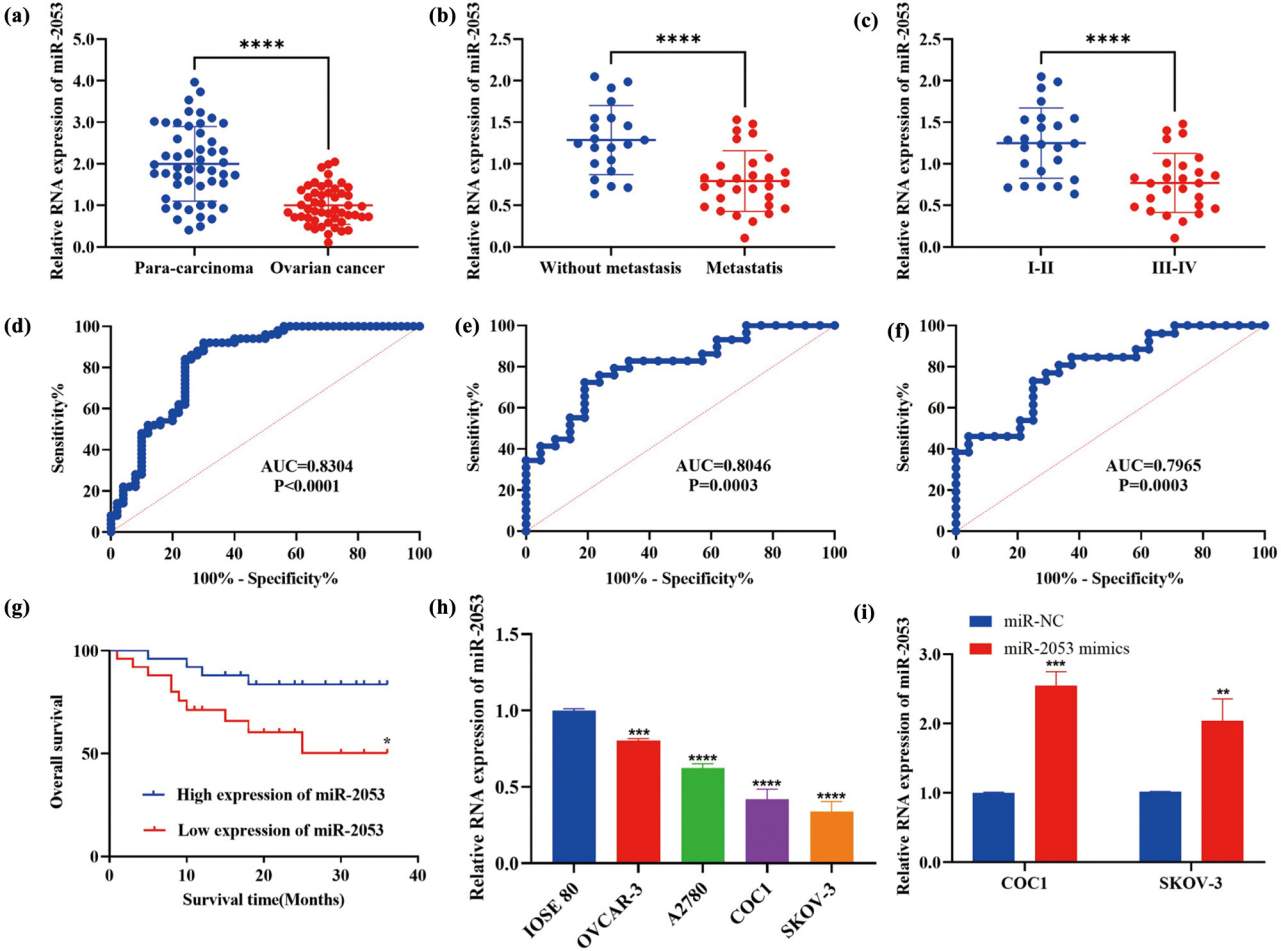
Downregulation of miR-2053 was detected in ovarian cancer tissues. (a) The levels of miR-2053 were assessed in 50 ovarian cancer specimens and matched adjacent normal tissues. (b) miR-2053 levels were evaluated in patients with and without metastasis. (c) miR-2053 expression was examined in ovarian cancer with different grades. (d–f) ROC curves were generated for data analysis, and the expression profile of miR-2053 showed high AUC values on differentiating ovarian cancer and non-cancerous tissues, patients with and without metastasis, and cases with low and high grades. (g) Overall survival was poorer in patients with low miR-2053 expression. (h) miR-2053 levels were decreased in ovarian cancer cells. (i) Transfection efficiency of miR-2053 mimics was confirmed by RT-qPCR. The experiments were performed in triplicates. *P* < 0.05; ^**^ *P* < 0.01; ^***^ *P* < 0.001; ^****^ *P* < 0.0001. AUC, area under the curve. NC, negative control.

The expression of miR-2053 was examined in 50 ovarian cancer samples and paired non-cancerous tissues. The levels of miR-2053 were remarkably reduced in ovarian cancer specimens compared to normal control (Figure 1a). In addition, miR-2053 expression was notably decreased in ovarian cancer patients with metastasis than the ones without metastasis (Figure 1b). Furthermore, the levels of miR-2053 were significantly downregulated in ovarian cancer cases with advanced grade (III–IV) compared grade I–II (Figure 1c). Moreover, results of ROC curves suggested that miR-2053 expression exhibited high AUC values on distinguishing ovarian cancer and adjacent normal tissues, cases with and without metastasis, and patients with higher and lower grade of tumour (Figure 1d–f). In addition, ovarian cancer patients with lower levels of miR-2053 showed poorer overall survival rate (log-rank test; Figure 1g) compared to the high expression group. Reduction of miR-2053 levels was also detected in ovarian cancer cells compared to the control (Figure 1h). The expression of miR-2053 was most significantly downregulated in COC1 and SKOV-3 cells; thus, these two cell lines were used for future experiments. All in all, the expression of miR-2053 was decreased in ovarian cancer specimens, which could be associated with tumour development. To further investigate the effects of miR-2053 on COC1 and SKOV-3 cells, miR-2053-transfected cells were used in the function study. Transfection efficiencies were assessed by RT-qPCR (Figure 1I).

### 3.2 Upregulation of miR-2053 suppresses the proliferation of ovarian cancer cells, while cell apoptosis was promoted

**Figure 2:**
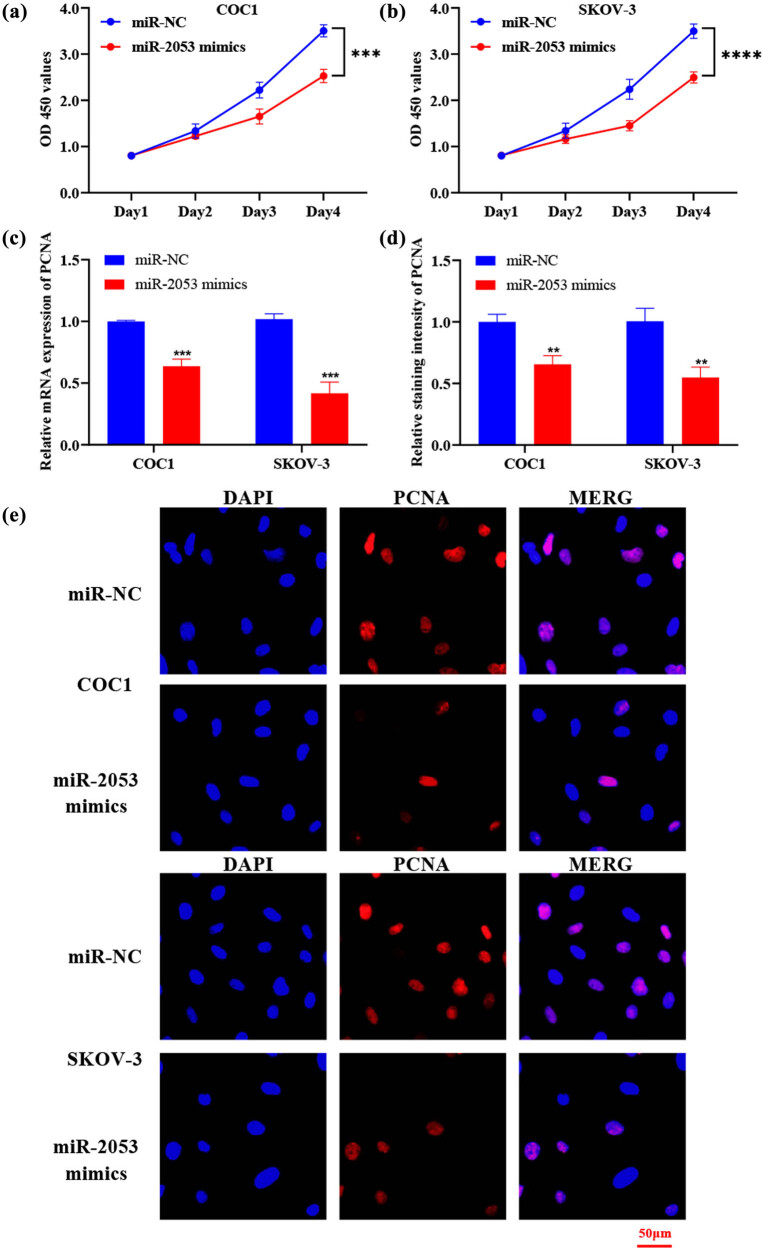
miR-2053 mimics inhibited the growth of ovarian cancer cells. (a and b) Cell proliferative activity was decreased by the transfection with miR-2053 mimics. (c) The mRNA levels of PCNA were reduced in cells treated with miR-2053 mimics. (d and e) The intensities of PCNA staining were decreased after the treatment with miR-2053 mimics. The experiments were performed in triplicates. ^**^ *P* < 0.01; ^***^ *P* < 0.001. NC, negative control.

**Figure 3:**
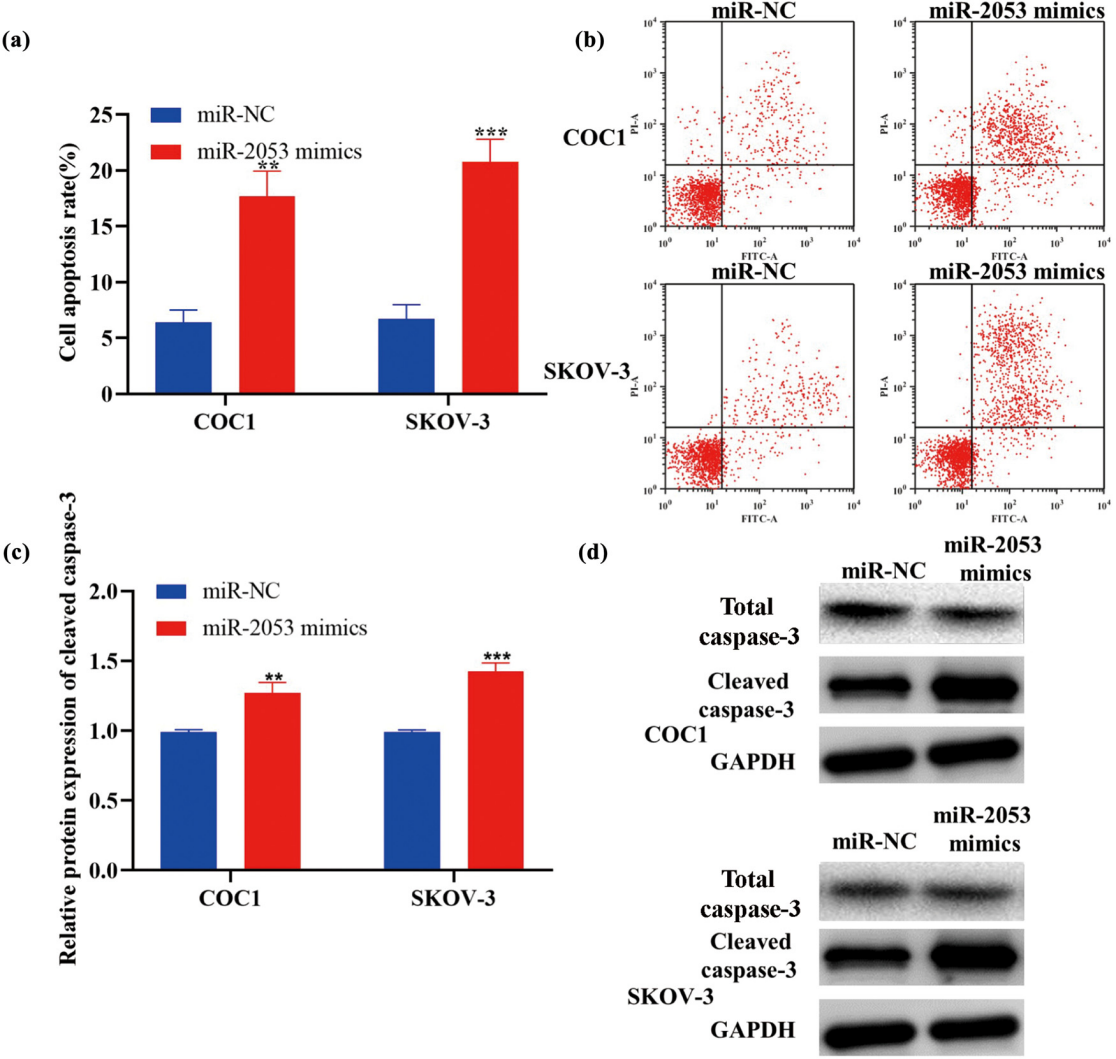
The apoptosis of ovarian cancer cells was enhanced by miR-2053 mimics. (a and b) Cell apoptotic rates were increased following the transfection with miR-2053 mimics. (c and d) The levels of cleaved caspase-3 were elevated in cells treated with miR-2053 mimics.The experiments were performed in triplicates. ^**^ *P* < 0.01; ^***^ *P* < 0.001. NC, negative control.

In our results, the CCK-8 assay revealed inhibited proliferation in ovarian cancer cells treated with miR-2053 mimics (Figure 2a and b). Furthermore, the levels of cell proliferation marker PCNA were examined in the transfected cells using RT-qPCR and immunofluorescence staining. The results indicated that the levels of PCNA were reduced in cells transfected with miR-2053 mimics (Figure 2c–e). In addition, flow cytometry suggested that cell apoptotic rate was enhanced following the transfection with miR-2053 mimics (Figure 3a and b). In consistent with these data, the levels of apoptosis-associated molecule cleaved caspase-3 were also increased in cells treated with miR-2053 mimics (Figure 3c and d). Taken together, our findings revealed that miR-2053 mimics were able to suppress the growth of ovarian cancer cells.

### 3.3 Migration and invasion of ovarian cancer cells are inhibited by miR-2053 mimics

**Figure 4:**
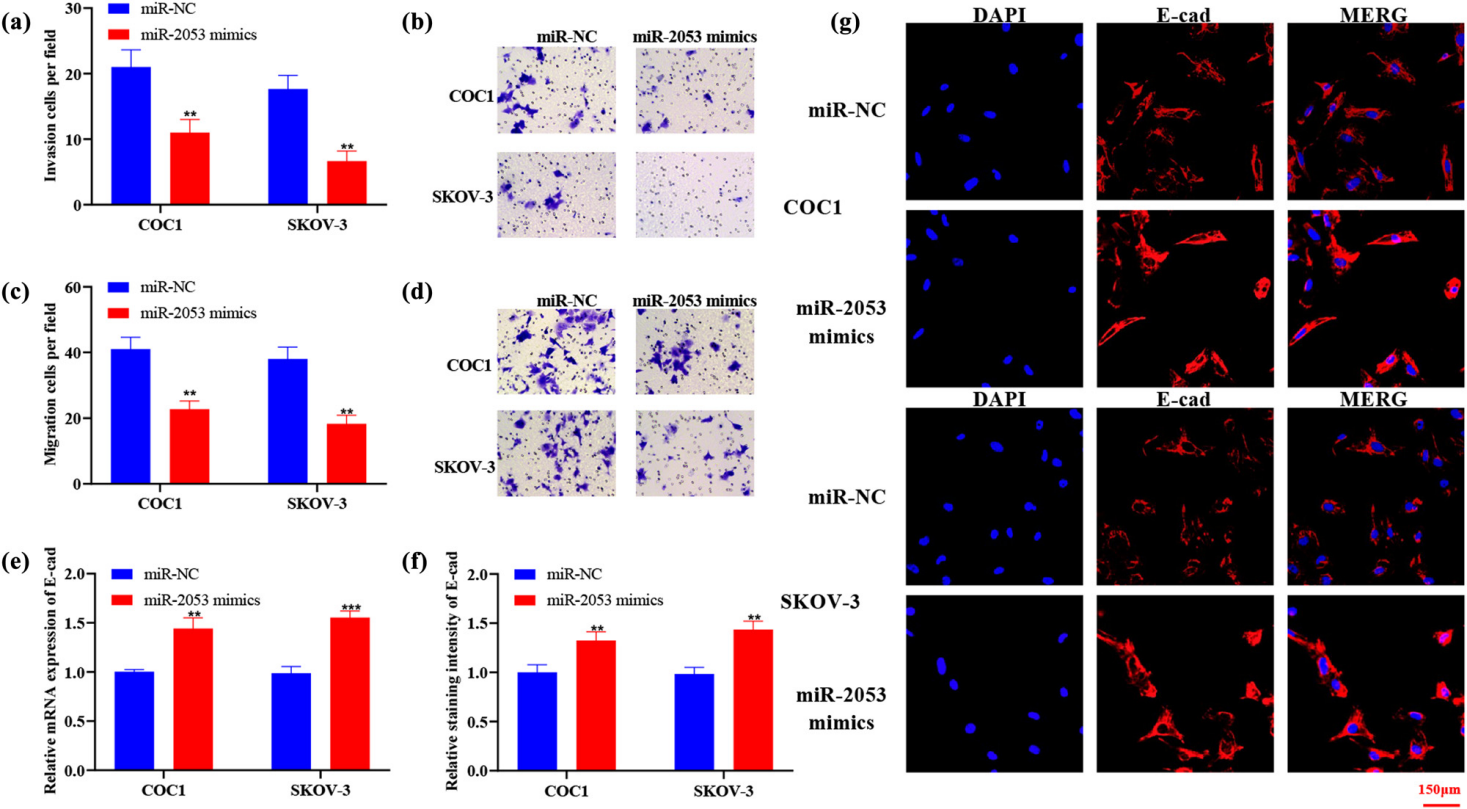
The migration and invasion of ovarian cancer cells were suppressed by miR-2053 mimics. (a–d) Cell migratory and invasive activities were examined using Transwell assay. (e–g) The mRNA and protein levels of E-cad were elevated after the transfection with miR-2053 mimics. The experiments were performed in triplicates. ^**^ *P* < 0.01; ^***^ *P* < 0.001. NC, negative control.

To further elucidate the influences of miR-2053 on the biological behaviour changes of ovarian cancer cells, cell migration/invasion was assessed. Transwell assay suggested that the migratory and invasive activities of both COC1 and SKOV-3 cells were suppressed by the transfection with miR-2053 mimics (Figure 4a–d). In addition, the expression levels of migration/invasion marker E-cad were examined in transfected cells. Both mRNA and protein levels of E-cad were increased in ovarian cancer cells treated with miR-2053 mimics (Figure 4e–g). In summary, these results suggested that upregulation of miR-2053 could suppress the migration/invasion of ovarian cancer cells.

### 3.4 SOX4 could be a promising target of miR-2053 in ovarian cancer

**Figure 5:**
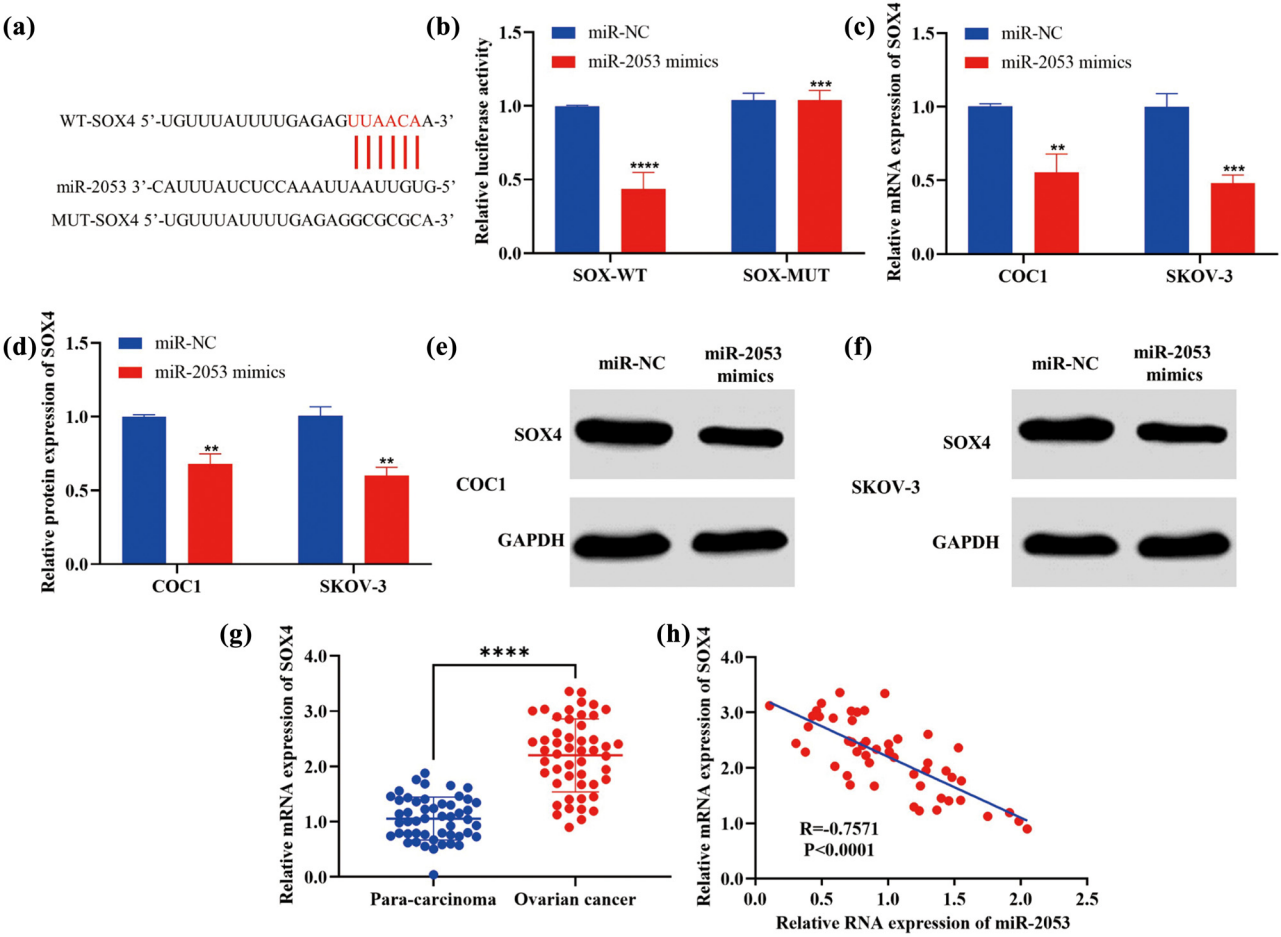
SOX4 could be a novel target gene of miR-2053 in ovarian cancer. (a) Bioinformatics analysis predicted the binding sites between miR-2053 and SOX4. (b) The interaction of miR-2053 and SOX4 was confirmed by luciferase reporter assay. (c) mRNA levels of SOX4 were decreased in cells transfected with miR-2053 mimics. (d–f) Western blotting indicated a reduction in SOX4 protein after the treatment with miR-2053 mimics. (g) SOX4 expression was enhanced in ovarian cancer tissues. (h) A negative correlation between miR-2053 and SOX4 levels was revealed. The experiments were performed in triplicates. ^**^ *P* < 0.01, ^***^ *P* < 0.001, ^****^ *P* < 0.0001. MUT, mutant; NC, negative control. WT, wildtype.

To investigate the detailed functions of miR-2053 in ovarian cancer and identify its downstream molecules, bioinformatic analysis was carried out. The 3′-UTR of SOX4 carried a highly conserved binding sequence of miR-2053 (Figure 5a). Furthermore, the luciferase reporter assay confirmed the interaction between miR-2053 and SOX4, as miR-2053 mimics notably reduced the luciferase activity of reporters containing the WT SOX4-binding sequence but not the mutant control (Figure 5b). Moreover, RT-qPCR results revealed the reduction of SOX4 mRNA in ovarian cancer cells following the treatment with miR-2053 mimics (Figure 5c). In addition, the protein levels of SOX4 were decreased in cells transfected with miR-2053 mimics (Figure 5d–f). Furthermore, the expression profile of SOX4 was evaluated in clinical samples, and the levels of SOX4 were remarkably elevated in ovarian cancer tissues compared to para-carcinoma controls (Figure 5g). In addition, a negative correlation between miR-2053 and SOX4 was revealed using Pearson’s correlation analysis (Figure 5h). Our data suggested that SOX4 was a novel downstream molecule of miR-2053 in ovarian cancer.

### 3.5 Alterations in ovarian cancer cells caused by miR-2053 inhibitors were enhanced by overexpressed SOX4, and the effects of miR-2053 mimics were abolished by SOX4 overexpression

**Figure 6:**
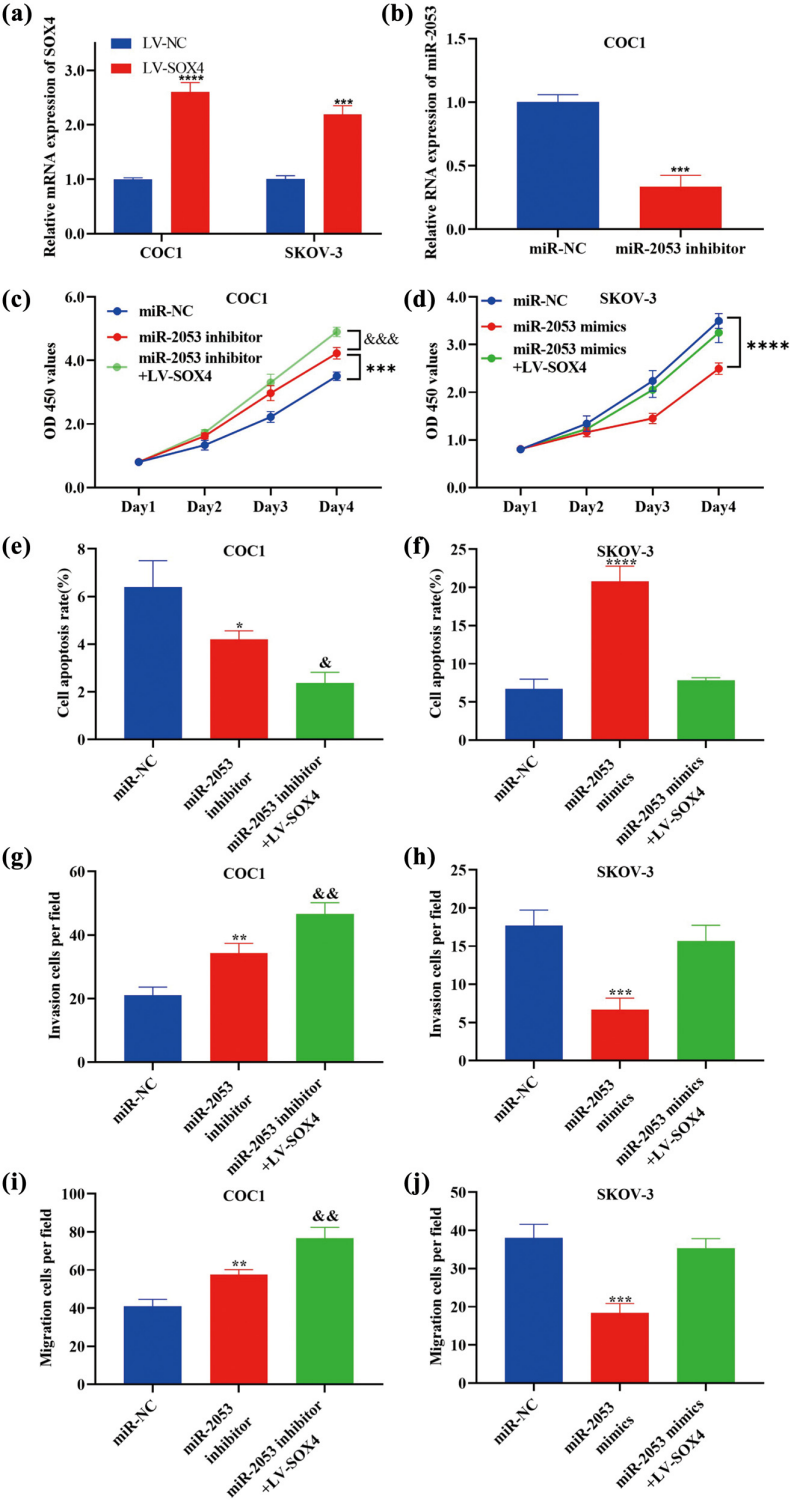
Biological behaviour alterations in ovarian cells caused by miR-2053 mimics and inhibitors were strengthened and reversed by LV-SOX4, respectively. (a and b) Transfection efficiencies of miR-2053 inhibitors and LV-SOX4 in ovarian cancer cells were examined by RT-qPCR. (c) Proliferation was promoted in cells transfected with miR-2053 inhibitors, which was enhanced by overexpressed SOX4. (d) The proliferative ability of ovarian cancer cells was suppressed by miR-2053 mimics, and these effects were reversed by LV-SOX4. (e) Cell apoptotic rate was decreased after the treatment with miR-2053 inhibitors, which was strengthened by LV-SOX4. (f) Cell apoptosis was enhanced following the transfection with miR-2053 mimics, and the influences were abrogated by LV-SOX4. (g and i) Cell migration and invasion were elevated in cells treated with miR-2053 inhibitors, which were enhanced by overexpressed SOX4. (h and j) Cell migratory and invasive activities were inhibited after the transfection with miR-2053 mimics, and these effects were abolished by LV-SOX4. The experiments were performed in triplicates. ^*^ *P* < 0.05; ^**^ *P* < 0.01; ^***^ *P* < 0.001; ^****^ *P* < 0.0001 vs LV-NC/miR-NC; ^&^ *P* < 0.05; ^&&^ *P* < 0.01 vs miR-2053 mimics/inhibitors. NC, negative control.

To perform further function study, ovarian cells were transfected with LV-SOX4 or miR-2053 inhibitors, and the efficiencies were examined by RT-qPCR (Figure 6a and b). Moreover, cell proliferation was enhanced following the transfection of miR-2053 inhibitors, and the effects were strengthened by LV-SOX4 (Figure 6c). Vice versa, proliferative activity was inhibited in cells treated with miR-2053 mimics, which was abrogated by LV-SOX4 (Figure 6d). In addition, flow cytometry revealed the reduction of cell apoptosis rate following the transfection with miR-2053 inhibitor, and these influences were enhanced by LV-SOX4 (Figure 6e). Moreover, cell apoptosis was promoted in cells treated with miR-2053 mimics, which was reversed by the transfection with LV-SOX4 (Figure 6f). In addition, cell migratory and invasive activity were increased after the treatment with miR-2053 inhibitors, which were enhanced by LV-SOX4 (Figure 6g and i). Vice versa, migration and invasion were suppressed in cells transfected with miR-2053 mimics, and these effects were reversed by LV-SOX4 (Figure 6h and j). In summary, the alterations in biological behaviours of ovarian cells induced by miR-2053 inhibitors and mimics were strengthened and abrogated by overexpressed SOX4, respectively. Our findings revealed the involvement of SOX4 in miR-2053-modulated ovarian cancer cells growth and metastasis, indicating that miR-2053/SOX4 signalling could be associated with the development of ovarian cancer.

## 4 Discussion

Impaired expression levels of miRNAs have been found in numerous types of cancer [13,14,15,16,17]. A previous study has also revealed the potential roles of miR-2053 as a key regulator during tumour progression in hepatocellular and oesophageal carcinoma [17,18]. In this study, the downregulation of miR-2053 was detected in ovarian cancer samples and cells. Overexpressed miR-2053 was able to suppress the proliferation, migration, and invasion of COC1 and SKOV-3 cells, whereas cell apoptotic rate was enhanced by miR-2053 mimics. These findings revealed the essential roles of miR-2053 as a novel tumour suppressor in ovarian cancer. Similarly, previous studies have also indicated that other miRNAs including miR-802 and miR-450a were putative suppressors of tumour progression in ovarian cancer [19,20].

In addition, our results revealed that SOX4 is a promising target molecule of miR-2053 in ovarian cancer. Moreover, upregulation of SOX4 was detected in ovarian cancer tissues, where its expression levels were negatively correlated with miR-2053. Furthermore, the alterations induced by miR-2053 inhibitors/mimics were enhanced/reversed following the co-transfection with LV-SOX4. SOX4 is a transcription factor, and it is a member of the SRY-related high mobility group box [21]. Recently, the association between SOX4 dysregulation and tumour progression has been reported [22,23,24,25,26,27]. For example, SOX4 could modulate the invasion of bladder cancer cells by suppressing WNT5 [22]. Upregulation of SOX4 is also associated with tumour development in nasopharyngeal carcinoma [23]. SOX4 could affect epithelial–mesenchymal transition in prostate, gastric, and breast cancers [24,25,26]. In summary, these findings revealed the important roles of miR-2053 and its novel target SOX4 in tumourigenesis of ovarian cancer. There are some limitations of this study. For instance, the protein level of SOX4 in the specimens could be determined.

To our knowledge, this is the first study investigating the involvement of miR-2053 during the tumour development in ovarian cancer. Our data suggested that miR-2053 was downregulated in ovarian cancer, and it could promote the growth of ovarian cancer cells. In addition, SOX4 was revealed as a novel target of miR-2053 in ovarian cancer. Upregulation of SOX4 was found in ovarian cancer specimens, where its expression was inversely correlated with miR-2053 levels. Moreover, biological behaviour changes in ovarian cancer cells caused by miR-2053 inhibitors were strengthened by overexpressed SOX4, whereas the effects of miR-2053 mimics were reversed by transfection with LV-SOX4. Taken together, miR-2053 together with its downstream molecule SOX4 are key regulators of cell proliferation, apoptosis, migration, and invasion in ovarian cancer. More importantly, miR-2053/SOX4 axis could be associated with tumour development in ovarian cancer and may be a novel candidate for future targeted treatment for this disease.
